# Upregulated expression of LOX is a novel independent prognostic marker of worse outcome in gastric cancer patients after curative surgery

**DOI:** 10.3892/ol.2012.1092

**Published:** 2012-12-27

**Authors:** QING ZHANG, XIAO-SHUN JIN, ZHONG-YIN YANG, MIN WEI, XIAO-CHENG ZHU, PING WANG, BING-YA LIU, QIN-LONG GU

**Affiliations:** 1 Department of General Surgery, Shanghai Institute of Digestive Surgery, Ruijin Hospital, Shanghai Jiao Tong University School of Medicine, Shanghai 200025;; 2 Jiangsu Key Laboratory of Biological Cancer Therapy, Xuzhou Medical College, Xuzhou 221002;; 3 Department of Pathology, Shanghai Ninth People’s Hospital, Shanghai Jiao Tong University School of Medicine, Shanghai 200011, P.R. China

**Keywords:** gastric cancer, LOX gene, tissue microarray, prognostic marker

## Abstract

Lysyl oxidase (LOX) initiates the enzymatic stage of collagen and elastin cross-linking. It also has intracellular functions involved in the regulation of cell differentiation, motility/migration and gene transcription. Aberrant expression of the LOX gene has been reported in multiple tumors. However, the correlation of its expression with clinicopathological parameters and its prognostic significance in gastric cancer remains largely unknown. In order to address this problem, total RNA of paired tissue samples (n=10) and a tissue microarray containing 161 paired tissues from patients with gastric cancers at different stages were collected. Quantitative real-time PCR and immunochemistry assay were conducted to investigate the expression of LOX. Based on the results, LOX mRNA was increased in gastric cancer tissues compared with the adjacent normal mucosa. Immunohistochemical detection revealed that expression of LOX was associated with depth of tumor invasion (P<0.05), lymph node status (P<0.05), TNM stage (P<0.05) and survival (P<0.05). Cox regression analysis revealed that positive expression of LOX (P=0.026) was an independent prognostic marker for survival in patients with gastric cancer.

## Introduction

The lysyl oxidase (LOX) gene family comprises five members which act as extracellular modulating enzymes; LOX, LOXL, LOXL2, LOXL3 and LOXL4 (1). The first identified and the more studied member of this family is LOX. The human LOX gene which spans across 15 kb of genomic DNA is located on chromosome 5 (5q23.3–31.2) and is comprised of seven exons that encode a 417-amino acid protein. LOX is synthesized as a 48-kDa preproprotein (preproLOX) which includes a 21-amino acid signal sequence at the amino terminus (2,3). PreproLOX is N-glycosylated and secreted from the cell as a catalytically inactive 50-kDa proenzyme protein (proLOX). ProLOX is subsequently cleaved into a catalytically mature 32-kDa protein (LOX) and a 180-kDa LOX-PP (1). The amino terminus of LOX contains the most unique sequence, while the carboxy terminus is highly conserved among its family members and is responsible for catalytic activity. The carboxy terminus contains a copper-binding site, lysyl tyrosyl quinine cofactor binding residues, a catalytically active site, a cytokine receptor and growth factor receptor-like domain (2,3).

LOX is a copper-dependent amine oxidase that maintains the covalent cross-linking of collagens and elastin in extra-cellular matrices, which is essential for normal function of connective tissue, embryonic development and adult tissue remodeling (4). Aberrant LOX expression or enzymatic activity is correlated with certain diseases, including cutis laxa, Menkes’ syndrome, spontaneous coronary artery dissection (5–7), atherosclerosis, scleroderma, liver cirrhosis and senile plaque formation in Alzheimer’s and non-Alzheimer’s dementia (8–10).

Recent studies have demonstrated that LOX has intracellular functions involved in the regulation of cell differentiation, motility/migration, gene transcription and cell adhesion. The aberrant LOX expression and activity that have been observed in various cancerous tissues and neoplastic cell lines are of interest (11). The role of LOX in in cancer has been controversial, due to both down- and upregulation of LOX in tumor tissues and cancer cell lines, which have been found in initial studies, suggesting a dual role for LOX as a tumor suppressor, as well as a metastasis-promoting gene (11–13).

Gastric cancer is one of the most common types of tumors and remains the second leading cause of cancer mortality in the world although the diagnosis and treatment of such patients have improved (14). Surgical resection is the main treatment modality and is able to cure patients with early-stage cancer. However, the survival rate of patients with advanced resectable gastric cancer remains poor, despite new treatment strategies, such as perioperative chemotherapy (15) and adjuvant chemoradiation (16).

Currently, numerous gastric cancer patients are diagnosed when the tumor is at an unresectable stage. For these patients, systemic chemotherapy is the main treatment option as it is able to prolong survival without impacting on quality of life. Certain single agents and combinations are effective in the treatment of suchmetastatic disease, but the survival of patients with advanced gastric cancer treated with palliative chemotherapy remains low. New therapies are therefore urgently needed.

At present, the TNM stage, tumor differentiation and histological classification are the primary criteria for predicting the clinical outcome in gastric cancer. As a highly heterogeneous tumor, prognosis in gastric cancer, however, often varies among patients with the same clinicopathological parameters in practice. Therefore, additional classification parameters need to be defined in addition to the TNM and the classic pathological characteristics of the tumor in order to better identify the biological subsets of this disease. Biological prognostic factors are often derived from the genetic process, which is thought to represent a crucial step to gastric cancer. Some of these potential prognostic factors may also be predictive of response to therapy as they are a molecular target either to chemotherapeutics or to biological/targeted therapies (17). With the aid of microarray technology, the identification of LOX as a potential modulator of tumorigenesis and/or metastatic tumor progression was carried out in this study.

## Materials and methods

### Patients and tissue samples

This study was approved by the Research Ethics Committee of Ruijin Hospital, Shanghai Jiao Tong University School of Medicine, China. Written informed consent was obtained from all the patients enrolled in this study. All specimens were handled and made anonymous according to the ethical and legal standards.

### Tissue specimens for real-time quantitative reverse transcription polymerase chain reaction (qRT-PCR)

Fresh specimens for qRT-PCR were obtained from 10 patients who underwent surgery for gastric cancer between March and May 2010 at the Department of Surgery, Ruijin Hospital, Shanghai Jiao Tong University School of Medicine. Grossly visible normal and cancerous portions of the specimens were snap-frozen in liquid nitrogen and stored at −85°C. None of the patients had received radiotherapy or chemotherapy prior to surgery.

### Tissue specimens and microarray construction

A total of 161 patients who had undergone curative surgery for gastric cancer at Ruijin Hospital, Shanghai Jiao Tong University School of Medicine, between January 2002 and December 2003 were enrolled in the study. Patient-derived specimens were collected and archived under protocols approved by the Institutional Review Boards of Shanghai Jiao Tong University. The group was composed of 107 males and 54 females with a mean age of 57 (range, 28–80) years at the time of surgery. There were 63 cases at stage I, 39 at stage II, 46 at stage III and 13 at stage IV. The diagnoses were confirmed by two pathologists, and the tumor grade and stage classifications were assigned according to the International Union Against Cancer guidelines (18). None of the patients had received radiotherapy or chemotherapy prior to surgery. Patients with advanced gastric cancer received standard chemotherapeutic protocols, including 5-fluorouracil postoperatively, according to the NCCN Practice Guidelines for Gastric Cancer (19). All patients were subjected to close follow-up observation. The follow-up deadline was October 2010.

For tissue microarray (TMA) construction, formalin-fixed, paraffin-embedded samples obtained from the above 161 patients that contained primary tumors and adjacent normal mucosa were retrieved from the archives of the Department of Pathology in Ruijin Hospital. Hematoxylin and eosin (H&E)-stained slides were screened for tumor tissue and non-cancerous tissue adjacent to the tumor (≥2 cm from the tumor). Representative areas of tissue were established and 2.0-mm diameter cores were punched from the paraffin blocks. Two cores from each tumor and paired normal tissue (≥2 cm from the tumor) were arrayed next to each other to ensure similar reaction conditions. All specimens were examined by two pathologists to prevent bias. Tumor and normal mucosa morphology on the arrays were validated as having high accordance with the whole archived section. TMA slides were constructed and made in collaboration with Shanghai Biochip (Shanghai, China).

### Real-time qPCR

Total RNA in 10 paired, frozen primary gastric cancer tissues and adjacent normal mucosa were extracted according to the manufacturer’s instructions (Life Technologies, Carlsbad, CA, USA), and then 0.5 *μ*g RNA was reverse transcribed into cDNA using a PrimerScript™ RT reagent kit (Takara Bio Inc., Dalian, China). qPCR was performed in a 10-ml total reaction mixture. Quantitative LOX mRNA levels were assessed using ABI 7500 real-time PCR System (Applied Biosystems, Carlsbad, CA, USA) with a Master Mix kit (Takara Bio Inc.) according to the manufacturer’s instructions. GAPDH was used as an internal control. Each real-time PCR was repeated 3 times and the 2^−ΔΔCt^ method was used for normalization with GAPDH as a reference gene. Gene expression with a ratio of >2 was considered to be upregulated. The sequences for qRT-PCR primers were as follows: GAPDH forward, 5′-TGTTGCCATCAATGACCCCTT-3′; GAPDH; reverse, 5′-CTCCACGACGTACTCAGCG-3′; LOX forward, 5′-CACAGGACATCATGCGTATGC-3′; LOX reverse, 5′-CCACTTCAGAACACCAGGCAC-3′.

### Immunohistochemistry

The TMA sections were deparaffinized in xylene and rehydrated in graded series of ethanols followed by heat-induced epitope retrieval in citrate buffer (pH 6.0). LOX expression was detected using a primary antibody against LOX (anti-LOX antibody, rabbit polyclonal to LOX, 1/300; Abcam, Cambridge, MA, USA). After incubation with a biotinylated secondary antibody and DAB (Dako, Carpinteria, CA, USA), the slides were rinsed and counterstained with Mayer’s hematoxylin. Staining was scored by two independent investigators without knowledge of patient outcomes according to the staining intensity and extent as described previously (20). To evaluate LOX expression, immuostaining was classified into four groups according to both intensity and extent. The proportion of cell protein expression was categorized as follows: 0, 0% immunopositive cells; 1, <10% positive cells; 2, 11–50% positive cells; 3, 51–75% positive cells; and 4, >75% positive cells. The staining intensity was categorized by relative intensity as follows: 0, negative; 1, weak; 3, moderate; 4, strong. The proportion and intensity scores were then multiplied to obtain a total score. To obtain final statistical results, scores ≤2 were considered as negative, while scores of 3–4 were considered as (+), scores of 5–8 as (++) and scores of 9–16 as (+++).

### Statistical analysis

Statistical analysis was performed using SPSS 17.0 statistical software (Chicago, IL, USA). Correlation was assessed with the Spearman Rho correlation coefficient and Pearson Chi-square test. Kaplan-Meier survival curves were generated and survival data were analyzed with the log-rank test and Cox proportional hazards regression. P<0.05 was considered to indicate a statistically significant result.

## Results

### Upregulation of LOX expression in primary gastric cancer tissues compared with adjacent normal mucosa

**Figure 1:**
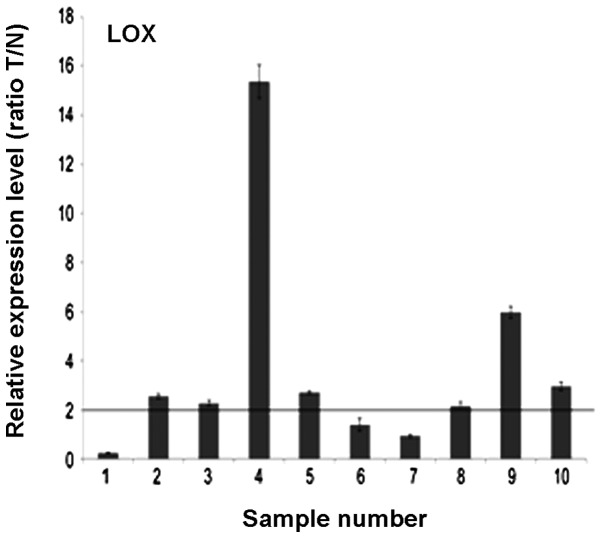
LOX mRNA expression was significantly higher in the primary gastric carcinoma tissues (70 vs. 30%, P<0.05). Relative expression of LOX mRNA in fresh gastric cancer tissues compared with normal tissues on qRT-PCR. All data were normalized using GAPDH as a reference gene. The consistency of the qRT-PCR starting template requires that the Ct-value be within 1. A normalized ratio of >2 on the Y-axis was considered to indicate gene expression upregulation; X-axis shows the specimen number. The black line corresponds to a Y-value of 2, showing the expression upregulation of the HOXC6 gene in specimen nos. 2, 3, 4,5, 8, 9 and 10. qRT-PCR, real-time quantitative reverse transcription polymerase chain reaction; LOX, lysyl oxidase.

Of the 10 paired cases used for the evaluation of LOX mRNA and protein expression, 7 (70%) gastric cancer tissues showed at least a four-fold increase in LOX mRNA level compared with the adjacent non-cancerous mucosa. The difference in LOX mRNA expression was significant (70 vs 30%; P<0.05; Fig. 1).

### Association of LOX TMA immunohistochemical staining with patient clinicopathological parameters

**Figure 2:**
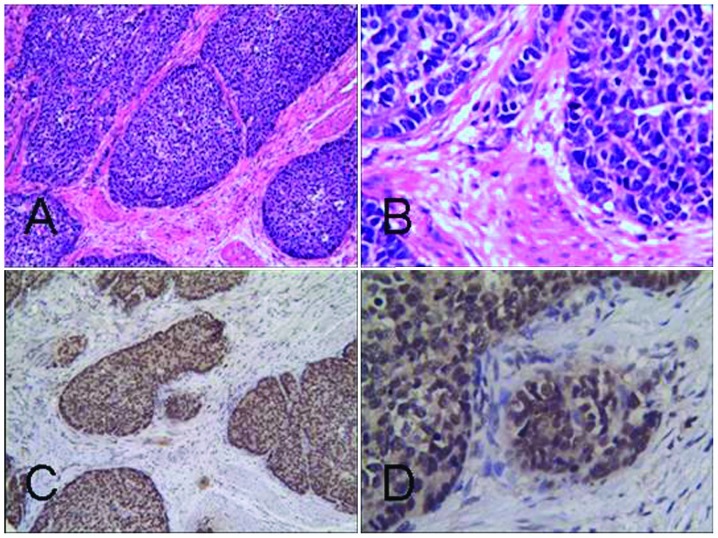

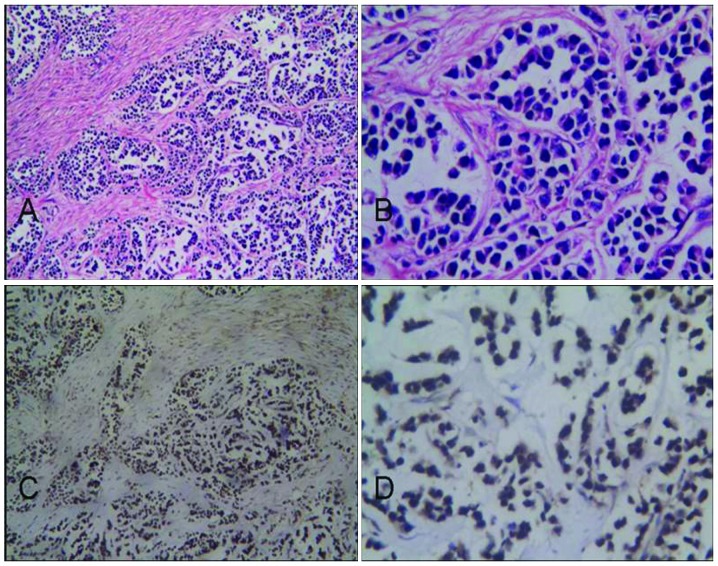
(A) Expression of LOX in primary gastric carcinoma poorly differentiated adenocarcinoma H&E staining: (a) magnification, ×100; (b) magnifcation, ×400; immunohistochemical staining: (c) magnification, ×100; (d) magnification, ×400. (B) Expression of HOXC6 in primary gastric carcinoma. Intermediate-differentiated adenocarcinoma H&E staining: (a) magnification, ×100; (b) magnification, ×400; immunohistochemical staining: (c) magnification, ×100; (d) magnification, ×400. LOX, lysyl oxidase; H&E, hematoxylin and eosin.

The expression of LOX was predominantly localized in the cytoplasm and nuclei of tumor cells. Of the 161 TMA specimens, expression of LOX was observed in 68 (42.2%) of the tumors. Of these, 54 cases had a score of +, 6 cases had a score of ++ and 8 cases had a score of +++. Meanwhile 20 (12.4%) of the adjacent noncancerous mucosa displayed positive staining. The difference in LOX expression between tumor cells and adjacent noncancerous mucosa was significant (42.2 vs. 12.4%, P<0.05; Fig. 2). Associations between clinicopathological factors and LOX expression are summarized in Table I. Increased LOX expression was significantly correlated with the depth of tumor invasion, lymph node status and TNM stage (P<0.05; Table I).

### Survival analysis and prognostic significance of LOX expression

**Figure 3:**
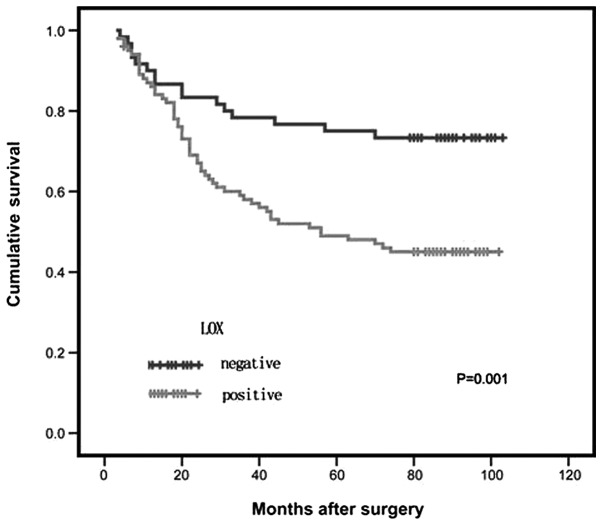
Univariate analysis of LOX protein expression with reference to overall survival (P<0.05). Survival curve of patients with gastric cancer with regard to LOX expression. Patients with positive LOX expression had a lower overall survival rate than patients with negative expression (P<0.05). LOX, lysyl oxidase.

**Figure 4:**
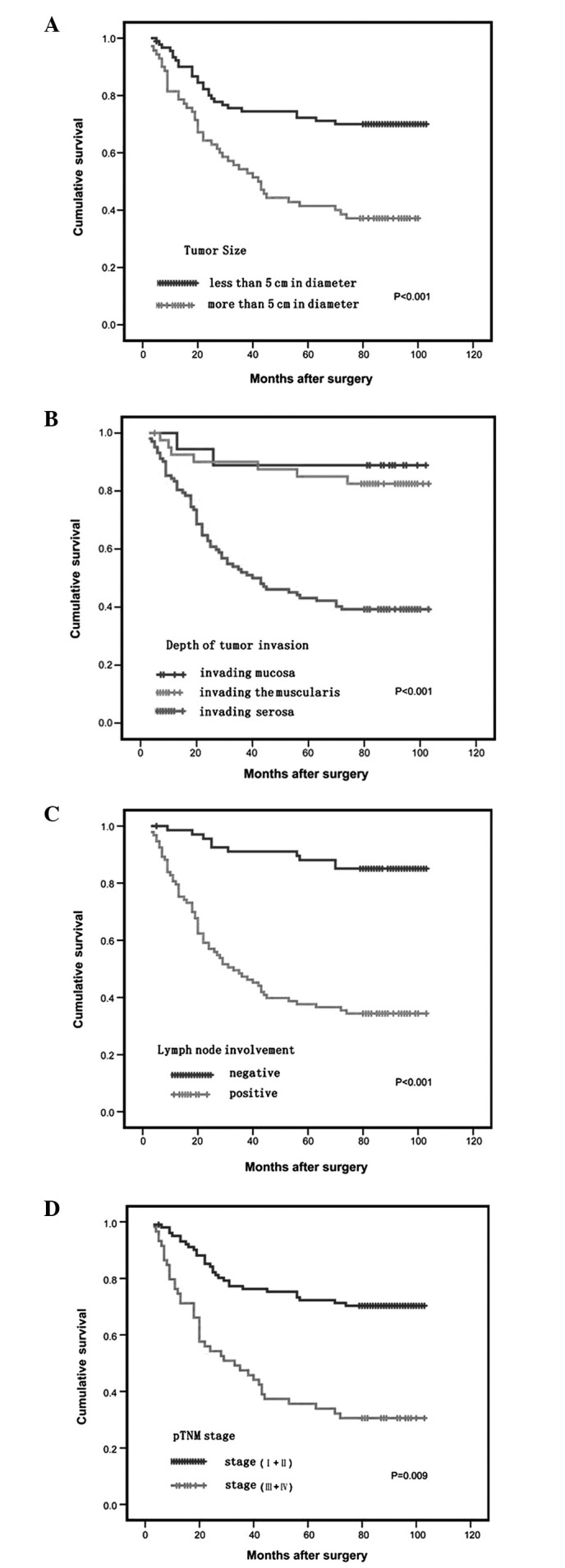
Univariate analysis of (A) tumor diameter (P<0.001), (B) depth of tumor invasion (P<0.001), (C) lymph node status (P<0.001) and (D) TNM stage (P<0.05) with reference to survival.

Kaplan-Meier curves showed that patients with positive LOX expression had a lower overall survival rate than the group with negative LOX expression (P<0.05; Fig. 3). As expected, the established clinical parameters of the tumor size, lymph node status, depth of tumor invasion and TNM stage significantly affected survival (P<0.05; Fig. 4).

In the Cox multiple regression analysis, depth of tumor invasion, histological differentiation, lymph node status and LOX expression independently significantly affected survival (Table II).

## Discussion

Currently, the TNM stage is the most frequently used for predicting prognosisfor gastric cancer. This classification system takes into account the depth of invasion of gastric wall (T), the involvement of lymph nodes (N) and the presence of distant metastasis (M). In the present study, the results of univariate analysis showed that tumor size, depth of invasion, involvement of lymph nodes and TNM stage are associated with the prognosis in gastric cancer, which was consistent with the results of previous studies (18).

LOX was initially reported as a copper-dependent amine oxidase responsible for the catalysis of collagen and elastin cross-linking within the extracellular matrix. However, previous studies have shown that LOX may have intracellular functions, including the regulation of cell differentiation, motility/migration and gene transcription (11,21). Since LOX protein structure and function are so complex and involve vital biological processes, such as cell movement, signal transduction and gene regulation, it is evident that aberrant regulation of LOX may lead to tumorigenesis and tumor progression (11).

Neoplastic transformation occurs as a result of genetic and epigenetic alterations in signalling pathways that mediate cell growth, cell cycle arrest and apoptosis. Genetic alterations may occur through mutational activation (e.g., oncogenes), mutational inactivation and loss of heterozygosity (e.g., tumor suppressor genes), as well as epigenetically (e.g., methylation/ demethylation of CpG dinucleotides) (22). A decrease in LOX mRNA and/or protein has been observed in basal and squamous cell, bronchogenic, colon, esophageal, gastric, head and neck squamous cell, pancreatic and prostatic carcinomas, as well as melanoma (11). The lack of LOX protein in tumor cells originating from keratinocytes is clearly associated with human basal cell carcinoma and squamous cell carcinoma skin cancers. The inhibition of the LOX enzymatic activity in the skin equivalent model induces basement membrane disruption and deregulation of filaggrin and K10 expression of the dermis, preparing a phenotype favorable to tumor development. The loss of LOX protein adds another step towards invasion (23). As for the role of LOX in gastric cancer, Kaneda *et al*(12) found that loss of heterozygosity and promoter methylation of LOX were detected in 33% (9 of 27) and 27% (26 of 96) of gastric cancers, respectively, suggesting that LOX is a tumor suppressor gene inactivated by methylation in human gastric cancers. Methylation-associated silencing of LOH was also observed in lung, colon and ovarian cancer cell lines (11). However, in our study, upregulated expression of LOX mRNA and protein has been found in gastric cancer. This finding suggests that there may be another mechanism by which LOX is involved in gastric cancer progression.

Tumor cell invasion is a complex process that involves attachment to, degradation of and detachment from an extracellular matrix, and finally active migration away from the primary tumor (10). Similar to tumorigenesis, metastatic progression also emerges as a result of genetic and epigenetic alterations in pathways that mediate cell invasion, survival outside of the primary tumor microenvironment and colonization/growth at a distant organ site (24). Tumor metastasis may be influenced by both the immediate microenvironment (cell-cell or cell-matrix interactions) and the extended tumor microenvironment.

An increase in LOX mRNAand/or protein also has been observed in breast (25), head and neck squamous cell (25), prostatic (26) and clear cell renal cell carcinoma (27), compared with their normal or non-aggressive neoplastic counterparts. In these studies, the expression of high levels of LOX mRNA and/or protein was a poor prognostic factor and was associated with poorly differentiated, high-grade tumors, increased recurrence rates and decreased overall survival. In our study, we also found that LOX protein expression was significantly correlated with depth of tumor invasion, lymph nodes status, and TNM stage. Upregulated expression of LOX was an independent prognostic marker of a worse outcome in gastric cancer patients, in agreement with results from Wilgus *et al* in lung adenocarcinoma (28).

As a component of the extracellular matrix, LOX plays a role in facilitating tumor-stromal interactions that are important for tumor progression and metastasis. The role of LOX in tumor progression has been most extensively studied in breast cancer. In these studies, LOX was considered to be important for late-stage tumor progression to metastasis, but not for earlier stages involving tumor formation. LOX was identified to be upregulated in breast cancer cells with metastatic ability and to facilitate breast cancer cell migration and adhesion through the hydrogen peroxide-mediated regulation of the FAK/Src signaling pathway leading to downstream changes in cell adhesion and migration (13,25).

Our results showed that LOX staining was predominantly present in cytoplasm and nuclei of gastric cancer cell, it has been shown that extracellular LOX was able to enter into the cytosol and became concentrated in the nuclei of smooth muscle cells through an unknown mechanism (29).

There have been no published studies on the possible association between LOX expression and the clinicopathological features of gastric cancer. Our results revealed significant correlations between the upregulated expression of LOX and the depth of tumor invasion, lymph node status and TNM stage. In the Cox multiple regression analysis, the upregulated expression of LOX was an independent prognostic marker of a worse outcome in gastric cancer patients. The strong correlations suggested that LOX upregulation may promote tumor invasion and metastasis and that LOX may possibly be used as a biomarker to identify subsets of gastric cancer with a more aggressive phenotype. These preliminary findings need to be verified in a larger, prospective, controlled, clinical study. The mechanism by which LOX is involved in gastric cancer progression has not been well elucidated in this study.

## Figures and Tables

**Table I:** Correlation between expression of LOX protein with clinicopathological parameters of gastric cancer.

	Expression of LOX protein		
Clinicopathological parameters	Negative	Positive	P-value
Gender			
Male	58	49	0.238
Female	35	19	
Age (years)			
≤60	59	32	0.053
>60	34	36	
Differentiation			
Poor	62	48	0.612
Well+intermediate	31	20	
Tumor site			
Pylorus	61	45	0.795
Gastric corpus	24	19	
Gastric fundus	8	4	
Tumor size (in diameter)			
≤5 cm	59	32	0.053
>5 cm	34	36	
Depth of invasion			
Mucosa	16	2	0.005
Muscular layer	25	16	
Serosa	52	50	
Lymph node status			
LN0	47	21	0.015
LN1-3	46	47	
p-Stage			
I+II	65	37	0.049
III+IV	28	31	

LOX, lysyl oxidase.

**Table II:** Cox multiple regression analysis for overall survival after surgery.

Characteristic	HR	95% CI	P-value
Age (years)	1.264	0.757–2.110	0.370
Gender	1.201	0.714–2.021	0.490
Tumor site	1.238	0.851–1.801	0.265
Tumor size	1.526	0.860–2.705	0.148
Lymph node status	3.928	1.875–8.229	0.000
Depth of invasion	1.989	1.028–3.845	0.041
p-Stage	0.527	0.299–0.929	0.027
Differentiation	1.241	0.709–2.171	0.450
LOX expression	1.804	1.074–3.027	0.026

HR, hazards ratio; 95% CI, 95% confidence interval; LOX, lysyl oxidase.
